# Effects of breathing reeducation on cervical and pulmonary outcomes in patients with non specific chronic neck pain: A double blind randomized controlled trial

**DOI:** 10.1371/journal.pone.0273471

**Published:** 2022-08-25

**Authors:** Sahreen Anwar, Asadullah Arsalan, Hamayun Zafar, Ashfaq Ahmad, Asif Hanif

**Affiliations:** 1 Department of Physical Therapy, Independent Medical College Faisalabad, Faisalabad, Pakistan; 2 University Institute of Physical Therapy, University of Lahore, Lahore, Pakistan; 3 Department of Rehabilitation Sciences, College of Applied Medical Sciences, King Saud University, Riyadh, Saudi Arabia; 4 University Institute of Public Health, University of Lahore, Lahore, Pakistan; Prince Sattam Bin Abdulaziz University, College of Applied Medical Sciences, SAUDI ARABIA

## Abstract

**Objective:**

The purpose of this randomized controlled trial was to study effects of breathing reeducation in the treatment of patients with non specific chronic neck pain.

**Methods:**

A total of sixty eight eligible patients with chronic neck pain were randomly allocated to breathing reeducation (BR) group (n = 34) and routine physical therapy (RPT) group (n = 34). Clinical outcomes were neck pain measured through visual analogue score, cervical active range of motion through CROM device, strength of neck muscles through hand held dynamometer and endurance of neck muscles measured through craniocervical flexion test. The neck disability was measured through neck disability index (NDI) and pulmonary outcomes such as forced vital capacity (FVC), forced expiratory volume in one second (FEV1), and FEV1/FVC ratio were measured through Spirolab 4. The outcomes were assessed at baseline and at 4 and at 8 weeks from baseline.

**Results:**

There were significant improvements in the BR group compared with the RPT group (P = 0.002) for cervical flexion, extension (P = 0.029), endurance (P = 0.042), strength of neck flexors (P <0.001), neck extensors (P = 0.034). Likewise there was a significant change in NDI (P = 0.011), FEV1 (P = 0.045), FVC (P <0.001), and FEV1/FVC ratio (P <0.001) in the BR group compared with the RPT group. The cervical side flexion and rotation showed no significant difference in breathing reeducation group with p > 0.05.

**Conclusion:**

Breathing reeducation combined with routine physical therapy is an effective treatment in patients with non specific chronic neck pain.

**Trial registration:**

IRCT 20200226046623N1, https://www.irct.ir/trial/46240.

## Introduction

Chronic neck pain is one of the leading causes of disability imposing personal and socioeconomic burden throughout the world [1]. The unique anatomy of cervical spine and various muscular attachments not only contribute to cervical movements but they also play a vital role in head and neck stability [2], mastication [3], kinesthetic sensation for good posture [4] and rib cage movements for the respiration [5]. Thus any dysfunction of the cervical spine can affect these important functions of human movement. The prime contributing factor in non specific chronic neck pain is myogenic, as consistent pain for weeks results in muscular imbalance and according to a study an association exists between chronic neck pain and altered activation pattern of cervical and thoracic muscles [6].

It is well documented that in certain postures of cervical spine, some cervical muscles, especially the sternocleidomastoids and scaleni assume the role of accessory inspiratory muscles, and participate in rib’s elevation and thoracic stability [7]. The over activity of these muscles and inhibition of deep cervical muscles leads to shallow breathing, less expansion of rib cage, hypocapnia, anxiety and more pain [8]. Previous studies have shown that cervical spine’s hypo mobility, decreased neuromuscular strength and endurance of neck muscles, decreased cervical proprioception, and altered psychological state may influence respiratory mechanism [9–11]. According to a recent review, maximal breathing capacity, arterial blood gas pressure, strength of respiratory muscles and chest mechanics are affected in patients with chronic neck pain [12]. The strong neuromuscular interactions, well knitted cervicothoracic anatomy and biomechanical interactions of the cervical and thoracic spine lead to disturbance in normal respiratory mechanism which might result in respiratory dysfunction in subjects with non specific chronic neck pain [13].

Since the chronic neck pain can impart a negative effect on respiratory function of patients with long lasting neck pain, so a holistic treatment approach has been suggested to treat non specific chronic neck pain and its associated disorders [14, 15]. The primary focus of chronic pain treatment program is pain management and general relaxation; and breathing techniques are important part of this program [16]. There is an evidence regarding altered breathing patterns in chronic neck pain, and breathing reeducation has an immediate positive effect on reduction of cervical muscle over activity and respiratory functions [13, 17]. According to a previous study the use of feedback respiratory exercises resulted in significant differences in terms of sternocleidomastoid activity and NDI between control and experimental groups [18].

Despite of plenty of literature regarding treatment of non specific chronic neck pain focusing on a multimodal treatment approach there is a lack of evidence regarding scientific testing and implementation of new treatment methods. The studies focusing on treatment of non specific chronic neck pain may have incorporated strengthening or stretching of neck muscles with postural reeducation but, no evidence was found regarding incorporation of breathing reeducation in the long term treatment protocol. According to a previous study breathing retraining may improve pain, movement patterns and neck muscle activity in patients with chronic neck pain [17]. However, in the said study only immediate effects of 30 minute breathing retraining with three different methods were observed on pain, cervical ROM and neck muscle activity without any explanation that which method was more favor able. In another study long term effects of breathing reeducation were studied in patients with neck pain but only pain, cervical range of motion and chest expansion were evaluated and no pulmonary function was assessed [19].

There remains an immense need of well designed randomized clinical trials regarding role of breathing reeducation in non specific chronic neck pain, with cervical and pulmonary outcome measures to suggest this treatment as part of holistic treatment protocols. Thus, the aim of the present study was to explore the effects of breathing reeducation on chronic neck pain, cervical ROM, neck muscles endurance, strength and quality of life. Additionally pulmonary functions were evaluated based on the hypothesis that improving the breathing pattern will result in improved respiratory capacity which might correct the abnormal activation pattern of superficial neck muscles, improve their force exerting capacity and improve quality of life.

## Methods

### Study design

This was a parallel group double blind (patient and assessor blind) randomized controlled trial with 1:1 allocation ratio in two groups. The trial was prospectively registered in Iranian registry of clinical trials (IRCT 20200226046623N1). The trial was conducted according to the consolidated standards of reporting trial CONSORT guidelines [20]. After ethical approval from the University of Lahore IRB -UOL- FAHS /697/ 2020: 23 January 2020, the data were collected from the patients attending the Physiotherapy Department District Headquarter Hospital Faisalabad, Pakistan. Written informed consent was taken from each participant prior to the study.

### Sample size calculation

The sample of 68 (34 in each group) was taken, the sample size was calculated using following formula at 80% power of study and 95% confidence level. Sample size calculation was derived from T. Duymaz study [21].

$$n=\frac{\left\{(\delta_{1}{}^{2}+\delta_{2}{}^{2})\times (Z_{1-}\alpha_{/2}+Z_{1-\beta }{)}^{2}\right\}}{{\left|\mu_{2}-\mu_{1}\right|}^{2}}$$

Here, n = 34 in each group, Z _1−α/2_ = Standardized Level of significance = 95% = 1.96, Z1_−β_ = Power of test = 80% = 1.28, μ_1_ = Mean in control group = 3.32, μ_2_ = Mean in physical therapy treatment group = 3.85, δ_1_^2^ = standard deviation in control group = 0.38, δ_2_^2^ = standard deviation in physical therapy treatment group = 0.55.

### Inclusion and exclusion criteria

Sixty eight patients with non specific chronic neck pain were recruited from the Physiotherapy Department, District Headquarter Hospital Faisalabad, Pakistan during August 2020 to June 2021. A systematic strategy was opted for the recruitment of patients through advertisement with posters and social media. The Patients were screened for the following eligibility criteria (1) nonspecific neck pain (2) neck pain duration for more than 3 months (3) willingness to participate in the study and random allocation. The patients were excluded from the study due to the following reasons (1) upper cervical symptoms (dizziness) (2) post traumatic neck pain, pain due to disc lesion, spondylosis and neck pain of nerve root origin [22] (3) smoking (4) asthma [23] (5) depression [24] (6) clinically obese patients and patients with any spinal deformity such as scoliosis and kyphosis. These conditions were excluded due to possible influence on the outcome measures during assessment and intervention maneuvers.

### Randomization and masking

Randomization was done through sealed envelope method and patients were randomly divided in two groups, breathing group and routine physical therapy group in 1:1 ratio, by an independent administrator. All participants, clinicians, and outcome measure assessors were blinded to the randomization process. In addition, the participants and outcome measure assessors and participants were also blinded to the type of intervention.

### Intervention

In routine physical therapy group, 34 patients (20 males, 14 females), mean age 39.00 ± 4.90 years, received a treatment comprised of infrared radiation (IRR) and isometric exercises of the neck muscles. Patients were instructed to lie in prone position and IRR was applied for 10 minutes on cervical region, followed by isometric exercises for cervical muscles (flexors and extensors) in supine lying with 10 second hold and 20 repetitions. After that each patient was instructed to perform placebo breathing exercises for 15 minutes. It was unsupervised random shallow routine breathing. In breathing reeducation group there were 34 patients (20 males, 14 females), mean age 39.70 ± 5.55 years, which received both routine physical therapy treatment and supervised breathing exercises. The supervision was done by an experienced physical therapist with more than ten years of experience in musculoskeletal and cardiopulmonary physical therapy. The details of the intervention are given below:

The patients were instructed verbally to assume a semi-Fowler’s position (To keep the torso supported and abdominal wall relaxed) and perform diaphragmatic breathing. The instructions were given in a smooth monotonous voice to avoid any distress or anxiety to the patients. The Patients were instructed to place one hand below the anterior costal margin, on the rectus abdominis and the other hand on the belly/navel region, and inhale slowly and deeply through the nose, from functional residual capacity to total lung capacity with a three-second inspiratory hold. It was followed by an instruction to relax the shoulders, keep the upper chest quiet in order that the abdomen is raised a little. The Patients were then instructed to exhale slowly through the mouth up to five seconds. The breathing exercises were performed in 3 sets for 15 minutes, each set lasted for 3 minutes with a rest of 2 minutes between each set. In between the repetitions of the diaphragmatic breathing exercise, the patient was told to breathe normally [25].

To avoid dizziness patients were advised to refrain from breathing from the top of the chest–try to keep the chest still and just let air in by allowing the stomach to gently rise and fall. Breathe through the nose and allow three seconds as they breathe in and five seconds as they breathe out. The total treatment time for both groups was the same. Patients of both groups received the intervention five days a week for consecutive 8 weeks.

### Outcome measures

**Fig 1:**
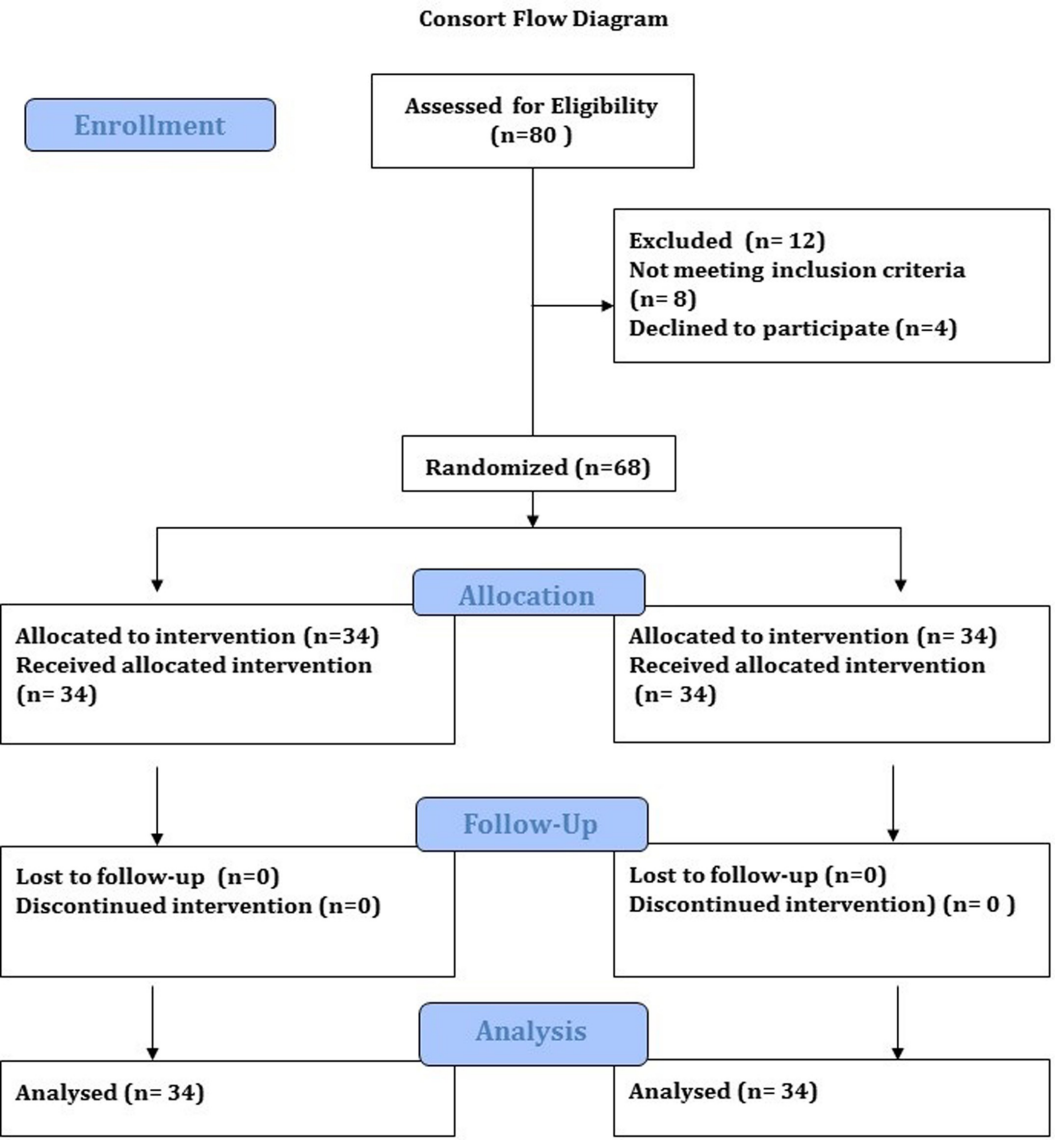
CONSORT flow diagram.

All outcomes measures were assessed by an assessor blind to treatment allocation. Pain and cervical ROM were assessed by visual analogue scale (VAS) [26] and CROM (basic) device by Performance Attainment Associates ^TM^(USA) respectively. Functional disability was measured through neck disability index NDI (Urdu Version) [27]. Cervical muscle endurance was measured through the craniocervical flexion test [28], and the strength of cervical flexors and extensors was measure by a special straight push pad of handheld dynamometer (Baseline Lite 200lb) which has shown to have a good interrater and intrarater reliability [29]. The pulmonary functions were measured through ‘Spirolab4” (MIR) in sitting position by a trained respiratory technician. The evidence of these tests is based on the official agreement between the American Thoracic Society and European Respiratory Society about the standardization of spirometery. Two pulmonary tests were performed (1) VC maneuver (2) FET (Forced expiratory technique) This maneuver included measurement of FEV1, FVC and FEV1/FVC ratio [30, 31]. The steps of the study are summarized in the CONSORT flow diagram (Fig 1).

### Data analysis

The data were analyzed using the SPSS 21.0 (SPSS Inc., Chicago, USA). The collected data were assessed fornormality using (Shapiro-Wilk test). For descriptive statistics, continuous variables were expressed as mean± SD (standard deviation), and for categorical variables frequencies and percentages were applied. For the inferential statistics, independent t-test and Mann–Whitney U test were used for between group comparison while Repeated measures ANOVA and Friedman test were applied for within group comparison at different time points (baseline, 4^th^ and 8^th^ week) and Bonferroni adjustment was used for pair wise comparison. For pair wise comparison of asymmetric variables at baseline, 4^th^ and 8^th^ week non parametric Wilcoxon signed rank test was used. The p value < 0.05 was considered as statistically significant for all analyzed data. All results were analyzed at 95% confidence interval.

## Results

During eight weeks of intervention program, all 68 participants with chronic neck pain completed the study. There were no significant differences between groups in the mean age and BMI (Body Mass Index), pain, NDI, endurance, strength of neck muscles and pulmonary functions. The average age, height, weight and body mass index of participants in breathing reeducation group and routine physical therapy group were 39.71±5.56 and 39.00±4.90 years, 156.35 ± 4.64 and 156.44 ± 3.66 meters, 65.15 ± 6.96 and 63.86 ± 6.09Kg, 27.01±1.67 and 26.67±1.65Kg/m^2 respectively. Whereas number of female and male participants was 14 (41.20%) and 14 (38.90%), and male participant was 20 (58.80%) and 20 (61.10%) in breathing reeducation group and routine physical therapy group respectively (Table 1).

**Table 1:** Baseline descriptive chracteristics (n = 68).

Demographic variables		Intervention	
	Categories / Units	Breathing reeducation (n = 34)	Routine physical Therapy (n = 34)
		Mean ±SD	
Age	Years	39.71±5.56	39.00±4.90
Height	Meter	156.35±4.64	156.44±3.66
Weight	Kg	65.15±6.96	63.86±6.09
Body Mass Index	Kg/m^2	27.01±1.67	26.67±1.65
Gender, n(%)	Male	20(58.80)	20(59.10)
	Female	14(41.20)	14(38.90)
**Total**		**34 (100)**	**34(100)**

SD = Standard Deviation, BMI = Body Mass Index,kg = Kilogram.

The results of repeated measure ANOVA for comparison in mean scores for cervical active range of motion (AROM) at different time points (4th and at 8th weeks) revealed a statistically significant difference with (F = 10.126; p < 0.001) (Table 2). At baseline, the mean cervical AROM scores in breathing reeducation group (BR) and routine physical therapy group (RPT) were flexion 36.59° ± 1.28, 36.25± 1.38 extension 50.26°±1.33, 50.39° ± 1.32, right lateral flexion 38.91°± 1.31, 38.92° ±1.36 left lateral flexion 39.91° ± 1.31, 39.70° ± 1.34 right rotation 50.47°±1.50, 50.86° ± 1.25 and left rotation 49.06° ± 1.92, 50.06° ± 1.98 respectively (Table 2).

**Table 2:** Between groups comparison for CAROM, NDI, neck flexors endurance and FEV1/FVC ratio.

Assessments	Intervention			F	P-Value	Partial Eta Square
	Breathing reeducation (n = 34)	Routine physical Therapy (n = 34)	Total (n = 68)			
	Mean ±SD					
Flexion Baseline (Degree)	36.59±1.28	36.25±1.38	36.41±1.30	10.126	0.002*	0.13
Flexion 4W (Degree)	39.41±1.50	38.44±1.86	38.91±1.80			
Flexion 8W (Degree)	42.15±1.79	40.05±2.18	41.07±2.20			
Extension Baseline (Degree)	50.26±1.33	50.39±1.32	50.33±1.32	4.99	0.029*	0.068
Extension 4W (Degree)	52.97±1.93	52.61±1.76	52.79±1.84			
Extension 8W (Degree)	56.76±2.77	54.28±2.01	55.49±2.70			
Right Flexion Baseline (Degree)	38.91±1.31	38.92±1.36	38.91±1.33	3.251	0.076	0.046
Right Flexion 4W (Degree)	41.53±1.65	40.94±1.72	41.23±1.55			
Right Flexion 8W (Degree)	43.59±1.65	42.42±1.50	42.99±1.67			
Left Flexion Baseline (Degree)	39.91±1.31	39.70±1.34	39.91±1.33	3.105	0.065	0.059
Left Flexion 4W (Degree)	42.53±1.65	41.11±1.42	41.23±1.55			
Left Flexion 8W (Degree)	44.59±1.65	42.92±1.50	42.99±1.67			
Right Rotation Baseline (degree)	50.47±1.50	50.86±1.25	50.67±1.38	0.693	0.408	0.011
Right Rotation 4W (degree)	53.06±2.17	52.86±1.78	52.96±1.97			
Right Rotation 8W (degree)	55.67±2.47	54.42±2.23	55.03±2.42			
Left Rotation Baseline (degree)	49.06±1.92	50.06±1.98	49.58±2.00	0.01	0.921	0.012
Left Rotation 4W (degree)	52.06±2.39	51.89±1.98	51.97±2.17			
Left Rotation 8W (degree)	54.50±2.62	53.53±2.17	54.00±2.43			
NDI Score at Baseline	13.41±1.58	13.14±1.76	13.27±1.67	6.857	0.011*	0.092
NDI Score at 4W	09.68±1.70	10.72±1.39	10.21±1.62			
NDI Score at 8W	06.56±1.93	8.36±1.87	07.49±2.09			
Endurance Neck Flexors at Baseline (mmHg)	16.56±1.08	16.75±1.48	16.66±1.30	4.308	0.042*	0.06
Endurance Neck Flexors at 4W (mmHg)	18.97±1.24	18.47±1.75	18.71±1.53			
Endurance Neck Flexors at 8W (mmHg)	21.97±1.60	20.14±2.06	21.03±2.06			
FEV1/FVC Ratio at Baseline (%)	64.41±1.86	64.31±2.32	64.36±2.09	15.631	<0.001*	0.187
FEV1/FVC Ratio at 4W (%)	67.88±1.81	66.31±2.35	67.07±2.23			
FEV1/FVC Ratio at 8W (%)	71.97±2.10	68.08±2.97	69.97±3.23			

“* “indicates the statistical significant results.

The p-value was calculated by repeated measure ANOVA.

CAROM: Cervical active range of motion, NDI: Neck disability index, FEV1: Forced expiratory volume in one second, FVC: Forced vital capacity, Ratio: FEV1/FVC ratio, w: week.

The results of repeated measure ANOVA for evaluation of change in mean NDI score and endurance of neck flexors at different time points were statistically significant with (F = 6.857; P = 0.011) and (F = 4.308; P = 0.042) respectively (Table 2). The results of repeated measure ANOVA for evaluation of change in mean score for FEV1/FVC ratio were also significant with (F = 15.631; P = 0.001) (Table 2).

The multivariate analysis from repeated measure ANOVA for the interaction effect of group by time (group *time) showed that there was a statistical significant differences between breathing reeducation group and routine physical therapy group at baseline, 4^th^ and 8^th^ week. Time*group effect was significant in breathing reeducation group according to the p values and partial eta square values where (P < 0.001, 0.203) for cervical flexion and (P < 0.001, 0.36) for cervical extension, whereas non significant for right and left sided flexion (P > 0.001, 0.184), (P > 0.001, 0.124) and right and left sided rotation (P>0.001, 0.238) (P > 0.001, 0.231) (Table 3).

**Table 3:** Main effect of time (Baseline, 4^th^ week and 8^th^ week) and interaction (Time*Group) for symmetric variables.

Variable	Effect	Value	F	P-Value	Partial Eta Squared
**Flexion (Degree)**	Time	0.114	259.15	<0.001*	0.886
	Time * Group	0.797	8.52	<0.001*	0.203
**Extension (Degree)**	Time	0.106	283.08	<0.001*	0.894
	Time * Group	0.64	18.85	<0.001*	0.36
**Right Flexion (Degree)**	Time	0.09	340.13	<0.001*	0.91
	Time * Group	0.816	7.54	0.001*	0.184
**Left Flexion (Degree)**	Time	0.086	220.11	<0.001*	0.855
	Time * Group	0.769	6.78	0.001*	0.124
**Right Rotation (Degree)**	Time	0.127	229.64	<0.001*	0.873
	Time * Group	0.762	10.44	<0.001*	0.238
**Left Rotation (Degree)**	Time	0.135	214.45	<0.001*	0.865
	Time * Group	0.769	10.07	<0.001*	0.231
**NDI Score**	Time	0.122	242.15	<0.001*	0.878
	Time * Group	0.801	8.3	0.001*	0.199
**Endurance Neck Flexor (mmHg)**	Time	0.091	335.4	<0.001*	0.909
	Time * Group	0.654	17.7	<0.001*	0.346
**FEV1/FVC Ratio (%)**	Time	0.157	179.82	<0.001*	0.843
	Time * Group	0.653	17.84	<0.001*	0.347

“* “indicates the statistical significant results.

The p-value was calculated by repeated measure ANOVA.

There was a significant group by time effect for NDI according to the p values and partial eta square values where (P < 0.001, 0.199), endurance of neck muscles (P <0.001, 0.346) and FEV1/FVC ratio (P < 0.001, 0.347) which showed that the NDI score showed more improvement in breathing reeducation group at 4^th^ and 8^th^ week as compared to routine physical therapy group. The endurance of cervical muscles and FEV1/FVC ratio showed statistically significant differences between breathing reeducation group and routine physical therapy group (Table 3). This difference is further explained with pair wise comparison of these outcome measures (Table 4).

**Table 4:** Pair wise comparison of CAROM, NDI, endurance of neck flexors and FEV1/FVC ratio at baseline, 4^th^ week and 8^th^ week.

Outcome Measures	Combinations at different timepoints	Mean Difference	P-value	95% Confidence Interval for Difference	
				Lower Bound	Upper Bound
Flexion (Degree)	Baseline-4th Week	-2.509	<0.001*	-2.825	-2.193
	Baseline-8th Week	-4.682	<0.001*	-5.203	-4.161
	4th Week-8th Week	-2.173	<0.001*	-2.558	-1.788
Extension (Degree)	Baseline-4th Week	-2.464	<0.001*	-2.789	-2.139
	Baseline-8th Week	-5.194	<0.001*	-5.744	-4.645
	4th Week-8th Week	-2.73	<0.001*	-3.171	-2.289
Right Flexion (Degree)	Baseline-4th Week	-2.323	<0.001*	-2.627	-2.019
	Baseline-8th Week	-4.088	<0.001*	-4.476	-3.701
	4th Week-8th Week	-1.766	<0.001*	-1.987	-1.544
Left Flexion (Degree)	Baseline-4th Week	-2.127	<0.001*	-2.427	-2.219
	Baseline-8th Week	-4.124	<0.001*	-4.176	-3.401
	4th Week-8th Week	-1.689	<0.001*	-1.287	-1.644
Right Rotation (Degree)	Baseline-4th Week	-2.294	<0.001*	-2.640	-1.949
	Baseline-8th Week	-4.381	<0.001*	-4.882	-3.879
	4th Week-8th Week	-2.087	<0.001*	-2.378	-1.795
Left Rotation (Degree)	Baseline-4th Week	-2.417	<0.001*	-2.815	-2.019
	Baseline-8th Week	-4.457	<0.001*	-4.993	-3.920
	4th Week-8th Week	-2.04	<0.001*	-2.331	-1.749
NDI Score	Baseline-4th Week	3.076	<0.001*	2.629	3.522
	Baseline-8th Week	5.815	<0.001*	5.170	6.460
	4th Week-8th Week	2.739	<0.001*	2.292	3.187
Endurance Neck flexors (mmHg)	Baseline-4th Week	-2.067	<0.001*	-2.307	-1.827
	Baseline-8th Week	-4.4	<0.001*	-4.820	-3.981
	4th Week-8th Week	-2.333	<0.001*	-2.635	-2.031
FEV1/FVC Ratio (%)	Baseline-4th Week	-2.735	<0.001*	-3.109	-2.361
	Baseline-8th Week	-5.668	<0.001*	-6.440	-4.896
	4th Week-8th Week	-2.933	<0.001*	-3.472	-2.394

“* “indicates the statistical significant results.

The p-value was calculated by repeated measure ANOVA.

FEV L/s: Force expiratory volume liter /second FVC: Force vital capacity.

Compared to the corresponding baseline values, the improvement in BR group was significantly greater than RPT group (intergroup mean difference for cervical flexion, -2.509; 95% CI,-2.85 to -2.193; P < 0.001at 4^th^ week and -4.682; 95% CI,-5.203 to-4.161; P < 0.001 at 8^th^ week, after Bonferroni correction). The (intergroup mean difference for extension from baseline to 4^th^ week was, -2.464; 95% CI,-2.789 to -2.139; P < 0.001 and -5.194; 95% CI,-5.744 to-4.645; P < 0.001 at 8^th^ week, after Bonferroni correction) (Table 4). For cervical right side flexion (intergroup mean difference at baseline and at 4^th^ week was, -2.323; 95% CI, -2.627 to -2.019; P < 0.001 and -4.088; 95% CI,-4.476 to-3.701; P < 0.001 at 8^th^ week, after Bonferroni correction). For Left side cervical flexion (intergroup mean difference at baseline and at 4^th^ week was, -2.127; 95% CI, -2.427 to -2.219; P < 0.001 and -4.124; 95% CI,-2.219 to-3.401; P < 0.001 at 8^th^ week, after Bonferroni correction). For right side rotation of the cervical spine (intergroup mean difference at baseline and at 4^th^ week was, -2.294; 95% CI, -2.640 to -1.949; P < 0.001 and -4.381; 95% CI,-4.882 to-3.879; P < 0.001 at 8^th^ week, after Bonferroni correction) and for left side cervical rotation(intergroup mean difference at baseline and at 4^th^ week was, -2.417; 95% CI, -2.815 to -2.019; P < 0.001 and -4.457; 95% CI,-4.993 to-3.920; P < 0.001 at 8^th^ week, after Bonferroni correction) (Table 4).

Compared to the corresponding baseline values, the improvement in BR group was significantly greater than RPT group (intergroup mean difference for NDI, 3.076; 95% CI, 2.629 to -3.522; P < 0.001at 4^th^ week and 5.815; 95% CI, 5.170 to 6.460; P < 0.001 at 8^th^ week, after Bonferroni correction) from baseline (Table 4). Compared to the corresponding baseline values, the improvement in BR group was significantly greater than RPT group (intergroup mean difference for endurance of neck flexors, -2.067; 95% CI,-2.307 to -1.827; P < 0.001at 4^th^ week and -4.4; 95% CI, -4.820 to -3.981; P < 0.001 at 8^th^ week, after Bonferroni correction) from baseline (Table 4).

Compared to the corresponding baseline values, the improvement in BR group was significantly greater than RPT group (intergroup mean difference for FEV1/FVC ratio, -2.735; 95% CI,-3.109 to -2.361; P < 0.001at 4^th^ week and -5.668; 95% CI, -6.440 to -4.896; P < 0.001 at 8^th^ week, after Bonferroni correction) (Table 4).

The between group comparison for pain through VAS (visual analogue scale) showed significant difference with P = 0.002* at 4^th^ week P <0.001* at 8^th^ week from the baseline. The between group comparison for strength of cervical flexors and extensors showed significant difference with P = 0.120, P = 0.436 at 4^th^ week and P <0.001* and P = 0.034 at 8^th^ week respectively from the baseline. The between group comparison for FVC showed significant difference with P = 0.012* at 4^th^ week P <0.001* at 8^th^ week from the baseline and the between group comparison for FEV1 showed significant difference with P = 0.267 at 4^th^ week P = 0.045* at 8^th^ week from the baseline (Table 5).

**Table 5:** Between and within group comparison for pain, strength of cervical flexors and extensors, FVC and FEV1 at baseline, 4^th^ week and 8^th^ week.

Assessments	Interventions		Mann-Whitney U	Z	P-Value^+^
	Breathing reeducation (n = 34)	Routine physical Therapy (n = 34)			
	Mean Rank				
Pain score at Baseline	36.24	34.81	587.00	-0.31	0.754
pain score at 4W	27.90	42.68	353.50	-3.16	0.002*
pain score at 8W	22.93	47.38	184.50	-5.18	<0.001*
**Chi Square value**	**68.00**	**69.39**			
**P-Value** ^**#**^	<0.001*	<0.001*			
Strength Flexion at Baseline	34.47	36.47	577.00	-0.43	0.665
Strength Flexion at 4W	39.18	32.03	487.00	-1.55	0.120
Strength Flexion at 8W	43.97	27.50	324.00	-3.48	<0.001*
**Chi Square value**	**65.79**	**69.39**			
**P-Value** ^**#**^	<0.001*	<0.001*			
Strength Neck Extensors at Baseline	34.47	36.47	577.00	-0.45	0.655
Strength Neck Extensors at 4W	37.26	33.83	552.00	-0.78	0.436
Strength Neck Extensors at 8W	40.50	30.78	442.00	-2.12	0.034*
**Chi Square value**	**67.51**	**71.51**			
**P-Value** ^#^	<0.001*	<0.001*			
Forced Vital Capacity at Baseline	37.82	33.31	533.00	-1.01	0.314
Forced Vital Capacity at 4W (Lt)	41.65	29.69	403.00	-2.52	0.012*
Forced Vital Capacity at 8W (Lt)	44.68	26.83	300.00	-3.71	<0.001*
**Chi Square value**	**68.00**	**72.00**			
**P-Value** ^#^	<0.001*	<0.001*			
One second Forced expiratory volume at Baseline	33.28	37.60	536.50	-1.10	0.271
One second Forced expiratory volume at 4W (Lt)	38.22	32.93	519.50	-1.11	0.267
One second Forced expiratory volume at 4W (Lt)	40.47	30.81	443.00	-2.01	0.045*
**Chi Square value**	**68.00**	**62.44**			
**P-Value** ^**#**^	<0.001*	<0.001*			

“* “indicates the statistical significant results.

“#” indicates the p-value calculated by non-parametric Friedman Test that is used for within group comparison at different time points (Baseline, 4^th^ week and 8^th^ week).

“+” indicates the p-value is calculated using Non- parametric Mann-Whitney U for between group comparison.

Chi square values are from Friedman’s chi square distribution table.

The pair wise comparison for the variables pain, strength of cervical flexors, FVC and FEV1 through Wilcoxon signed rank test at baseline, at 4t week and at 8^th^ week is shown in (Table 6).

**Table 6:** Pair wise comparison of pain, strength of cervical flexors and extensors, FVC and FEV1 at baseline, 4^th^ week and 8^th^ week.

		Intervention			
		Breathing reeducation (n = 34)		Routine physical Therapy (n = 34)	
		Z Score	P-Value	Z Score	P-Value
Pain score	4th Week - Baseline	-5.215	<0.001*	-5.357	<0.001*
	8th Week - Baseline	-5.179	<0.001*	-5.292	<0.001*
	8th Week - 4th Week	-5.17	<0.001*	-4.889	<0.001*
Strength of Neck Flexors	4th Week - Baseline	-5.114	<0.001*	-5.308	<0.001*
	8th Week - Baseline	-5.131	<0.001*	-5.312	<0.001*
	8th Week - 4th Week	-5.208	<0.001*	-5.523	<0.001*
Strength of Neck Extensors	4th Week - Baseline	-5.323	<0.001*	-5.385	<0.001*
	8th Week - Baseline	-5.192	<0.001*	-5.311	<0.001*
	8th Week - 4th Week	-5.224	<0.001*	-5.454	<0.001*
Forced Vital Capacity	4th Week - Baseline	-5.157	<0.001*	-4.941	<0.001*
	8th Week - Baseline	-5.121	<0.001*	-5.217	<0.001*
	8th Week - 4th Week	-5.188	<0.001*	-4.516	<0.001*
One second Forced expiratory volume	4th Week - Baseline	-5.1	<0.001*	-5.194	<0.001*
	8th Week - Baseline	-5.095	<0.001*	-5.173	<0.001*
	8th Week - 4th Week	-4.967	<0.001*	-4.518	<0.001*

The p-value was calculated by Wilcoxon signed Rank test.

## Discussion

The present study was aimed to compare the effects of breathing reeducation on pain, cervical range of motion, disability, strength of neck muscles, endurance of neck muscles and pulmonary functions in patients with chronic neck pain. Breathing reeducation with routine physical therapy showed significant improvement in cervical and pulmonary outcomes from baseline to eight weeks. The improvement in pain, cervical flexion and extension range of motion (AROM), cervical flexor strength and neck disability index was significant in breathing reeducation group as compared to routine physical therapy group. The pulmonary functions of the patients were significantly improved in breathing reeducation group.

The results of this study are persistent to a review study about effects of breathing exercises on pain, quality of life and pulmonary functions in patients with chronic low back pain [32]. In this study best available researches were explored and there was a moderate evidence for the effectiveness of breathing exercises on pain, quality of life and pulmonary function in patients with chronic back pain. The results of the present study are also comparable to a study in which immediate effects of breathing reeducation were observed in 36 subjects with chronic neck pain. In the said study pain, cervical range of motion and chest expansion were evaluated before and immediately after the intervention and statistically significant improvement was found in the treatment group [17]. In contrast our study aimed to find out effects of breathing reeducation for eight weeks and outcomes were measured at baseline, 4th and at 8^th^ week providing a more vast understanding about effects of breathing reeducation. In addition to pain and ROM, endurance, strength quality of life and pulmonary functions of patients with chronic neck pain were also assessed.

In a previous study effectiveness of neck stabilizing exercises combined with breathing reeducation exercises was assessed in 45 patients with stroke [33]. In the said study the effects of 30 minute exercise program was compared in two experimental and one control group. The conclusion of this study support our hypothesis as after six week evaluation only experimental group with combined regime of stabilizing exercises and breathing exercises showed improvement in neck flexors thickness, forced vital capacity and peak cough flow. In a cross sectional study on 44 neck pain patients and 31 healthy individuals, neck muscle strength was correlated to respiratory function of chronic neck pain patients i.e. more disability more dysfunction [34]. This correlation was evident in our study where combination of neck isometric exercises and breathing reeducation in patients with chronic neck pain resulted in improved strength of cervical muscles and improved pulmonary function.

Breathing reeducation is an effective regime to improve pulmonary functions of the patients with chronic neck and back pain [17, 32]. In a study on 40 healthy males (age 20–29 years) breathing exercises combined with upper extremity exercises resulted in significant improvement in FVC, whereas there were no significant intergroup differences in FEV1 and peak expiratory flow rate. The participants in the said study were divided in two groups, both groups received breathing exercises and the experimental group performed dynamic upper extremity exercises in addition to breathing exercises for four weeks [35]. The results of this study support our findings according to which the patients with chronic neck pain who were in breathing reeducation group showed significant improvement in FVC, FEV1 and FEV1/FVC ratio at four weeks and further improvement at eight weeks from the baseline. A recent study compared the effects of adding respiratory exercises in therapeutic routine for smart phone users with chronic neck pain and found it an effective treatment [36]. In that study 60 patients (aged 24.7±2.1 years) from both genders were divided equally in three groups, pain, muscle activity and respiratory parameters were measured before and at eight weeks of the treatment. There were significant improvements in the combined group compared with the therapeutic routine group (p = 0.03) for diaphragm muscle activation, (p = 0.03), neck erector spine activity (p = 0.04), respiratory balance (p = 0.04), and number of breaths (p = 0.02). The results of this study are consistent with our study where addition of breathing reeducation in routine physical therapy treatment resulted in improved pain, disability and pulmonary functions measured through spirometery. As compared to this study where respiratory parameters were measured through respiratory balance and number of breaths only, we used an authentic assessment approach measuring FVC, FEV1 and FEV1/FVC ratio through spirometery.

The present study is the first comprehensively designed clinical trial to investigate the efficacy of breathing reeducation with routine physical therapy in chronic neck pain patients. According to the results, breathing reeducation was found effective in improving cervical outcome measures, decreasing pain and disability level in chronic neck pain patients whereas pulmonary functions were also improved.

### Limitations of the study

There are a few limitations of the study. Only forced expiratory technique (FET) and vital capacity (VC) were measured to assess pulmonary functions to avoid lengthy measurement procedures as patients with chronic neck pain were involved in the study. However, maximum voluntary ventilation (MVV) and chest expansion should have been measured to have a more understanding about effect of breathing reeducation on pulmonary function. Provided the lack of meticulously designed clinical trials on effect of breathing reeducation in chronic neck pain patients, multicenter pragmatic trials are required for the generalizability of the present findings in the treatment of chronic neck pain. Moreover, we recommend categorization of participants on the basis of breathing dysfunction prior to the breathing reeducation so that more understanding of the effects of breathing intervention among various categories can be understood.

## Conclusion

Breathing reeducation combined with routine physical therapy treatment improves pain, cervical flexion and extension range of motion, endurance and strength of neck flexors in patients with chronic non specific neck pain moreover; it also improves disability, FVC, FEV1 and FEV1/FVC ratio in patients with chronic neck pain. Thus, breathing reeducation may be an effective regime to improve cervical and pulmonary outcomes in chronic neck pain patients.

## Supporting information

S1 Checklist Consolidated standards of reporting trials checklist. (DOC)

S1 File Study protocol. (DOCX)

S1 Data Study data. (XLSX)
