# Supplementary material for: Effects of breathing reeducation on cervical and pulmonary outcomes in patients with non specific chronic neck pain: A double blind randomized controlled trial
Source: PLoS One. 2022 Aug 25;17(8):e0273471. doi: 10.1371/journal.pone.0273471 (PMC9409509; doi:10.1371/journal.pone.0273471)
Supplement: S1 File — (DOCX) [file pone.0273471.s002.docx]

**A Research Proposal Entitled**

**Effects of breathing reeducation on cervical and pulmonary outcomes in patients with chronic neck pain: A randomized controlled trial.**

**Principal Investigator: Sahreen Anwar**

**INTRODUCTION**

Neck pain is one of the leading causes of disability ranked at number 4 according to the recent epidemiological study of Mayo clinic, and its annual prevalence is increasing by 30-50%[^1^](#_ENREF_1).According to global burden disease report published in 2015 more than a third of a billion had neck pain of more than 3 months duration[^2^](#_ENREF_2). Acute neck pain may get resolved within 4 weeks with or without any intervention but more than one third of population continue to suffer annually [^3^](#_ENREF_3). The causative factor of neck pain can be osteogenic, myogenic, or neurogenic but most of the time it is related to poor biomechanics and bad posture. The risk factors for chronic neck pain have been categorized into physical, neurophysiological and psychosocial[^4^](#_ENREF_4).

From Anatomical perspective Neck pain can be defined as “stiffness and/or pain felt dorsally inthe cervical region between the occipital condyles and the C7 vertebral prominence”[^5^](#_ENREF_5). Apart from many classifications of neck pain one classification is based on its duration as Acute or chronic depending on that how long it persists. According to IASP classification for ICD-11 published in 2019 non specific neck pain which persists more than three months and interferes with patient’s activities of daily living is labeled as chronic neck pain [^6^](#_ENREF_6)^,^ [^7^](#_ENREF_7).Neck pain due to its area of distress has a multidimensional impact and a number of disabilities can accompany neck pain such as cervicogenic headache, postural disturbances lack of sensorimotor control, respiratory dysfunction ,lack of psychosocial well being and altered head and eye movements[^8^](#_ENREF_8).

The major precipitating factor in chronic neck pain is myogenic as long term persistent pain leads to muscular imbalance where over activity is seen in superficial neck muscles while deep neck flexors are not fully activated. More specifically, in neck pain there is an inhibition of deep cervical flexors (longus colli, longus capitis), whereas superficial neck flexors (sternocleidomastoid, anterior scalene) present increased activation [^9^](#_ENREF_9). There is a reduced ability in chronic neck pain patients to relax their superficial neck flexors and extensors after activation[^10^](#_ENREF_10). The cervical spine enables performance of all movements including flexion, extension, lateral flexion and rotation. The complicated kinematics of the neck can influence the related muscles altering their force-length curve which can have an impact on their properties and ultimately their function.[^11^](#_ENREF_11)

The muscles of the cervical region can be divided in three discrete categories according to their anatomical position 1) muscles of the craniocervical region, 2) muscles typical of the cervical region and 3) muscles which coexist in both regions. Longus colli, sternocleidomastoid and anterior scalenus are the most important flexors of the neck. Sternocleidomastoid is a long strap-like muscle of the neck region and arises from two heads. Anterior scalenus is found deep to sternocleidomastoid and it is also found in pair. When one of them contracts it causes lateral flexion of the neck and rotation to the opposite side. When In case of the head and neck being fixed, the clavicle and manubriumsterni are elevated expanding the rib cage. This latter function indicates their usefulness as accessory muscles during inspiration[^12^](#_ENREF_12). Trapezius is a large, flat, superficial and triangular muscle which extends from the spine and skull to the pectoral girdle. Beside the other functions of the trapezius such as stabilization, retraction or lateral rotation of the scapula and elevation of the pectoral girdle, it also significantly contributes to neck motion. More specifically, when both of the trapezius act together they extend the neck and head, whereas when they act singularly they lead to lateral flexion of neck and head. The trapezius is also an accessory muscle of inspiration as it helps in the elevation of the thoracic cage.

Chronic neck pain accompanies a number of disorders but respiratory dysfunction has not been given importance in the usual clinical practice. In recent past there is a growing interest in the relation of respiratory dysfunction in chronic neck pain population. Respiration is a multidimensional phenomenon which is influenced by number of factors such as biomechanical, biochemical and social. Major factors reported in literature are (a) the decreased strength of deep neck flexors and extensors, (b) the hyperactivity and increased fatigability of superficial neck flexors, (c) the limitation of range of motion, (d) the decrease in proprioception and disturbances in neuromuscular control, (e) the existence of pain and (f) the psychosocial influence of dysfunction.More specifically the pain experienced by the patient also contributes to the respiratory dysfunction as noxious stimuli may increase respiration and drugs may inhibit it. Evidence about respiratory function of neck pain patients is also provided in a study by Nilsen et al (2007) aimed at investigating activation of the autonomous nervous system and pain after low-grade mental stress in patients with fibromyalgia and shoulder/neck pain.

Muscular imbalance,postural changes and segmental instability due to weakness of deep neck muscles may lead to thoracic spine instability and changes in rib cage mechanics. Changes in proprioceptive feed back, psychological influence of pain and resulting kinesophobia further exaggerates the dysfunction. Increased neck muscle fatigueabilty,decreased ROM due to pain further contribute to change in biomechanics of rib which finally leads to respiratory dysfunction.

After the sound reasoning for this clinically proven phenomenon there comes a need to solve this neglected aspect of chronic neck pain for patient’s well being.Many treatment options are used for the treatment of chronic neck pain such as opioid analgesics,electrotherapy,Manual therapy,physical exercises,alternative medicine such as acupuncture and needling but a small number of patients can get rid of it completely while rest of the patients continue to suffer through rest of their life. Individualized home exercises, laser, pulse electromagnetic treatment, strengthening exercises and proprioceptive exercises are suggested to have sufficient evidence to support their effectiveness on treating chronic neck pain[^13^](#_ENREF_13)^,^ [^14^](#_ENREF_14) .There is a great need for better treatment options asthat particularly deal with the “chronic” aspect of non specific neck pain as chronicity involves long term fascial and soft tissue adhesions resulting in increased dysfunction and disability. According to a meta analysis by co Riordan et al and clinical practice guidelines by American orthopedic association for treatment of chronic neck pain clinicians should use a multimodal treatment approach including ,mobilization of musculoskeletal structures, exercises focusing on endurance and strength of neck muscles ,aerobic exercises, and functional exercises[^15^](#_ENREF_15).

Despite of all the literature focusing on a multimodal treatment approach and calling for specific treatment methods to treat the particular pain provoking structural elements and associated dysfunctions there is a lack of evidence where new treatment methods have been implemented and tested scientifically.The studies may have incorporated strengthening and patient education,or strengthening or stretching maneuvers but no evidence was found regarding breathing education in the protocol [^16^](#_ENREF_16). In a study finding effectiveness of global posture reeducation breathing exercises were part of protocol but study was only conducted on females and outcome measuring tools were subjective only[^17^](#_ENREF_17) .Keeping in view the less explored treatment aspects of non specific chronic neck pain further studies incorporating specific on point techniques in the multimodal treatment approach are needed,so that patient can get rid of long spans of pain and live a healthy life. Moreover after the established evidence of respiratory dysfunction in non specific chronic neck pain it is necessary to explore effects of breathing reeducation as part of treatment approach to make patients pain free and improve their quality of life. When devising an appropriate rehabilitation plan for non specific chronic neck pain patient’s potential disturbances of respiratory function should be also taken into consideration. Further more in a recent systematic review by Kahlee et al investigation of the effectiveness of management of CNP on respiratory function is strongly suggested.[^18^](#_ENREF_18)

Thus the main purpose of this study is to investigate the effects of addingbreathing reeducation which has never been examined together along with strengthening exercise in chronic neck pain patients. The study aims at putting together the change in cervical and respiratory outcome measures after the implementation of breathing treatment protocol for non specific chronic neck pain patients.

**LITERATURE REVIEW**

Cervical spine consists of 7 cervical vertebrae and is functionally divided into upper cervical spine and lower cervical spine .The cervical spine has a complicated function as this region does not only consist of uncovertebral and zygapophysial joints, but also of atlantoaxial and atlanto-occipital articulations rendering the cervical kinematic. The 7^th^ cervical vertebra is relatively large in size and is in more resemblance to thoracic spine.[^19^](#_ENREF_19)

Cervical muscular system is composed of superficial or outer layer of muscles , able to produce large torques for movement production and a deep muscular system contributing to the stability of cervical spine.The long term improper muscular balance leads to greater activation of superficial muscles[^20^](#_ENREF_20)thus leading to greater fatigueabilty and even altered morphology[^21^](#_ENREF_21). Particular neck muscles such as sternocleidomastoids, trapezius and scaleni have dual role in neck movement and inspiration. Scaleni,particularly scalenus medius are active during quiet inspiration and contribute to breathing even when the pulmonary volumes increase is very small[^22^](#_ENREF_22), whereas sternocleidomastoids are relaxed during quiet breathing and are activated mainly during forced inspiration[^23^](#_ENREF_23). Although scaleni are more active and have a greater mechanical advantage, the greater mass of sternocleidomastoid leads both muscles to similar respiratory effects.Furthermore, muscles such as the inferior and superior oblique and rectus capitis muscles (anterior, lateralis, posterior major and posterior minor) have a more stabilizing role contributing to stability of cervical and thoracic spine as well .Any deficit in the function of both mobilizer and stabilizer muscles may directly or indirectly affect chest biomechanics with a consequential change of force-length curves and adaptive changes of respiratory muscles [^24^](#_ENREF_24). Deficits in function may be due topain, tightness, fatigue and kinesophobia.

Respiration is a specialized function and its variation depends on number of factors from anatomical buildup to age, gender ,lifestyle ,smoke exposure and metabolic diseases[^25^](#_ENREF_25). The close relation of cervical and thoracic spine in terms of location , fascial and ligamentous attachments the coordinated action of neck muscles as neck flexors and rotators along with being inspiratory muscles calls for influence of one area to another[^18^](#_ENREF_18). This inbuilt anatomical and biomechanical relation alongwith neuromuscular influence of the cervical muscles in respiration is the basis of the model developed by kapreli et al (2008) describing potential development of respiratory dysfunction in patients with chronic neck pain[^24^](#_ENREF_24). According to this model, the deficits accompanying chronic neck pain including reduced strength and endurance of neck muscles, altered cervical proprioception, reduced mobility of the cervical area, psychological states as well as pain by itself may directly influence respiratory muscles. The altered function is due to the common use of sternocleidomastoid, trapezius and scaleni or indirectly through a change in rib cage mechanics. These changes in parallel with the direct effects of pain on ventilation may finally lead patients with chronic neck pain to respiratory dysfunction.

Evidence about the existence of respiratory dysfunction in chronic neck pain is limited however following studies describe this phenomenon. A study conducted on chronic neck pain patients assuming forward head posture  demonstrated a strong association with decreased respiratory muscle strength in neck patients[^26^](#_ENREF_26). According to a review by Dimitradis et al about the evidence regarding respiratory dysfunction in neck pain patients maximal voluntary ventilation, strength of respiratory muscles, chest mechanics and partial pressure of arterial carbon dioxide are affected[^27^](#_ENREF_27). Patients with chronic neck pain can even reach up to the limit of hypocapnia i.e reduced partial pressure of carbon dioxide due to physical and psychological manifestation of chronic neck pain [^28^](#_ENREF_28). In another study to check the pulmonary functions of patients with chronic neck pain results suggested that patients do not have optimal pulmonary functions and they yielded significantly reduced vital capacity, FVC, expiratory reserve volume, and maximum voluntary ventilation (P < .05)[^29^](#_ENREF_29). The forward head posture assumed in chronic neck pain also leads to decreased respiratory parameters as proven in a study by kim et al( 2017) [^30^](#_ENREF_30).According to another study conducted in 2018 on healthy males by H zafar et al different head and neck posture influence respiratory function[^31^](#_ENREF_31).

Many treatments options are under practice for the treatment of arthrogenic and myogenic components of non specific chronic neck pain which usually place limit on mobility of cervical spine[^32^](#_ENREF_32) .Joint mobilization and manipulation is most widely used treatment to cure hypomobility and usually less focus is laid on the soft tissues and non mechanical elements accompanying cervical and thoracic movements.The release of myofascial adhesions is an important part of chronic neck and back pain as it has marked effects in restoring the mobility[^33^](#_ENREF_33).Current evidence regarding respiratory dysfunction in chronic neck pain suggests respiratory function, assessment and treatment should be part of routine physical therapy treatment [^5^](#_ENREF_5).In a study it was suggested that thoracic mobility and cervical muscle endurance should be improved in patients suffering from chronic neck pain[^34^](#_ENREF_34). In another study aimed to determine the effects of inspiratory muscle training on the relative respiratory muscle activity in healthy adults it was concluded that an accurate training focusing on deep breathing will be beneficial[^35^](#_ENREF_35).So incorporating a breathing reeducation intervention in the treatment of chronic neck pain patients produces significant effects in improving efficiency of SCM, Scaleni and trapezius[^36^](#_ENREF_36).In recent past many studies have provided strong evidence regarding nonspecific chronic neck pain and its relation to respiratory dysfunction but literature regarding treatment approach to benefit the chronic neck pain patients by improving their respiratory function is sparse[^27^](#_ENREF_27).There remains an immense need of randomized control trials with strong outcome measures to confirm the evidence base of such approach.

**PROBLEM STATEMENT**

Neck pain is one of the leading cause of disability and is ranked at number 4 in terms of overall burden [^37^](#_ENREF_37).Rates of recurrence of neck pain are high and females suffer more as compared to males [^38^](#_ENREF_38).There are many causativefactors from trauma ,disc pathology to idiopathic causes but postural disturbances due to certain occupations and increased gadget use in current era is leading to more prevalence of neck pain[^37^](#_ENREF_37). There is an increasing economic burden due to neck pain in terms of treatment cost, lost wages and medical insurances. In many countries economic burden due to neck pain is just second to back pain[^38^](#_ENREF_38). So there is a great need to find out the evidence based treatment options which can provide better management of the problem and prevent from undue expenses. According to literature review there is lot of studies conducted in recent past exploring the chronic neck pain and its associated effects on respiratory functions. Considering the results of these studies it is evident that there is association between chronic neck pain and respiratory dysfunction due to the associated musculoskeletal build up of cervical and thoracic region. However literature does not provide the treatment regimes which can benefit both of these problems, most studies aim at treating a single region at a time but are unable to explore the treatment effects in greater benefit. The wide research gap in exploring the non invasive conservative treatment options for neck pain as well as respiratory dysfunction associated with it calls for treatment options to cure these problems.

**OBJECTIVES**

- To compare the effects of breathing reeducation on clinical outcomes in non specific chronic neck pain.

**OPERATIONAL DEFINITIONS**

**Effects**: To assess changes in clinical and functional outcomes

**Clinical outcome**: Pain, Range of motion

**Functional outcome**: Disability, Endurance of cervical muscles involved in respiration, strength of cervical muscles involved in respiration.

**Chronic neck pain**:Non specific neck pain which persists more than 3 months[^39^](#_ENREF_39)

**NDI**: Neck disability index

**HYPOTHESIS**

**Null Hypothesis**: There is no difference in clinicaloutcomes with breathing reeducation in non specific chronic neck pain.

**Alternate Hypothesis**: There is difference in clinical outcomes with breathing reeducation in non specific chronic neck pain.

**MATERIAL AND METHODS**

**STUDY DESIGN**

The study design will be a double blinded randomized controlled trial.

**SETTINGS:**

This randomized clinical trial will be performed in the following location.

Department of physical therapy, District Headquarter Hospital Faisalabad

**DURATION**

18 Months after approval of synopsis from institutional review board.

**Sample Size**

The sample of 68 (34 in each group) will be taken, with adding 20% dropout rate n=(34+6)=40 Sample size is calculated using following formula at 80% power of study and 95% confidence level


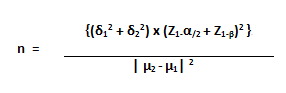


Here , n= 40 in each group

Z _1-α/2_ = Standardized Level of significance = 95% = 1.96

Z_1-β_= Power of test = 80% = 1.28

µ_1_= Mean in control group = 3.32

µ_2_= Mean in physical therapy treatment group = 3.85

δ_1_ ^2^ = standard deviation in control group = 0.38

δ_2_ ^2^ = standard deviationin physical therapy treatment group = 0.55

Duymaz T, Effect of physiotherapy on respiratory functions in patients with chronic neck pain 2019.Journal of clinical and analytical medicine DOI: 10.4328/JCAM.6123

**SAMPLING TECHNIQUE**

The purposive non probability sampling technique will be used to recruit the patients and random assignment of patients into both groups will be through sealed envelope method.

**SAMPLING CRITERIA**

**Inclusion criteria**

- Patients having non specific neck pain for more than 3 months
- Both genders
- Patients having no history of respiratory disease
- Patients having no history of antidepressant drug treatment
- Age between 25-50 years

**Exclusion criteria**

- Smokers
- Known history of depression
- Patients having any sort of Asthma
- Patients with upper cervical signs and symptoms
- Prolong sitting

**ETHICAL APPROVAL**

1. It will be obtained prior from the Ethical Committee of the University before study.
2. Written informed consent will be taken from all the patients.
3. All information and collected data will be kept confidential.
4. Participants will remain anonymous throughout the study.
5. The subject will be informed that there are no disadvantages or risk on the procedure of the study.
6. They will also be informed that they will be free to withdraw at any time during the process of the study.

**Data collection procedure**

A sample of 80 patients, aged 25-50years, will be recruited for this study. Chronic neck pain will be defined as pain lasting for more than 3 months. All the subjects will be informed about the purpose and nature of this study and their informed consent will be obtained before participation. The participants will be divided equally into two groups by sealed envelope method.

The examiner will complete a General Health Questionnaire for recording demographics and establishing eligibility criteria through interview with the participants.

A Symptoms and Pain History Questionnaire including body charts, symptomatology questions and Visual Analogue scales will also be allocated to the patients.

- Both groups will be having routine physical therapy treatment in form of infrared for 10 minutes at cervical area, neck isometrics for flexors, extensors, and side flexors in lying position. Group A will receive routine physical therapy treatment in form of infrared for 10 minutes, isometric exercises for flexor, extensor and side flexor of cervical spine in supine lying 20 repetitions with 10 second hold, Group B will receive supervised breathing exercises focusing on proper inhalation, exhalation and chest expansion for 15 minutes in addition to routine physical therapy treatment. Patients will undergo the intervention twice a week for consecutive 8 weeks. Outcome measures will be taken for musculoskeletal and respiratory elements on baseline and at 4^th^and 8^th^ week respectively. For musculoskeletal element pain and ROM will be assessed by using visual Analogue scale and CROM, functional disability will be measured through Neck disability index. Cervical muscle strength and endurance will be measured through isometric neck dynamometer and craniocervical flexion test respectively. For respiratory element spirometery will be used for assessing pulmonary volumes.
- **Breathing Exercise**

The method to teach diagphragmatic (or abdominal or belly) breathing is as follows:

- Assume a comfortable position, usually lying flat on your back with your knees bent up, in a quiet and calm environment
- Begin by relaxing your shoulders and arms
- Place one hand on your chest and the other hand on your belly/naval region
- Inhale slowly through your nose for 5-8 seconds
  - As you breathe in, your belly should rise and your lower ribs should expand outwards with minimal upper chest movement.
  - Exhale slowly through your mouth relaxing your chest wall and abdomen, usually for the same duration (or slightly longer) than your breath.

**Tools& Measurements**

**Body Composition**

Height (cm) and weight (kg) will be measured, to establish general baseline criteria for participants

**FOR Cervical element**

1. **VAS (visual Analogue score)**

It is a graph from 0 to 10; patients determined their pain intensity scores between 0 and 10. Zero represents no pain and 10 the most severe pain. So earning a higher score in this test means more pain. VAS is a 10 cm line where the participants are asked to mark the point on the line which best represents their pain intensity, always considering that the start of the line means no pain and the end of the line means the worst pain that someone can experience. According to VAS scores neck pain can be described as mild (0-30mm), moderate (30-60mm), moderately severe (60-80mm) and severe (80-100mm) (Collins et al, 1997; Konstantinovic et al, 2010).

1. **CROM(device**) **For Cervical ROM**

Cervical ROM will be measured in a sitting posture with a CROM device that combines inclinometers and magnets (CROM Basic, Performance Attainment Associates, Lindstrom, MN); this instrument has been shown to be reliable and valid for the measurement of CROM[^40^](#_ENREF_40).

1. **Neck Dynamometer for cervical muscle strength**

To check strength of cervical flexors, extensors ,side flexors **.**

**4.Craniocervical flexion test** (pressure biofeedback(Chattanooga Group, Hixson, TN, USA)for cervical muscle endurance

**5. Neck Disability Index (NDI)URDU**

The NDI has been developed by Vernon and Mior (1991) and is a 10-item questionnaire .NDI  is both reliableand validated tool for neck disability[^41^](#_ENREF_41)^,^ [^42^](#_ENREF_42). It consists of 10 sections punctuated from 0 to 5 each, and can determine the level of disability due to neck pain. The higher the score obtained, the greater neck pain interference in daily routine. Its scores can be categorized into five categories, which are: scores from 0-4 indicates no disability; 5-14 indicates mild disability, 15-24 moderate disability, and 25-34 severe disability.NDI is compatible with descriptors associated with the International Classification of Functioning and Health. In this study the Urdu version of NDI will be used,having good reliability and responsiveness by Urdu speaking patients[^43^](#_ENREF_43).

**FOR respiratory element**

**Lung Function Measurement**

All subjects will perform spirometry in which FET (Forced expiratory technique) will be performed .This maneuver will include measurement of FEV1, FVC, FEV1/FVC, .The evidence of this test is based on the official agreement between the American Thoracic Society and European Respiratory Society about the Standardization of Spirometry (Miller et al, 2005) . The acceptability criteria for these tests have been also analytically described by Ruppel (2009).

**List of Dependant variable**

| Pain | ROM | Disability | Strength of neck muscles | Endurance of neck muscles |
| --- | --- | --- | --- | --- |
|  | FET values |  |  |  |

**List of independent variable**

| Breathing Reeducation | Routine physical Therapy Treatment |
| --- | --- |

**Statistical Analysis**

Data will be analyzed through SPSS version 22. Descriptive statistics in form of frequency, percentage and bar chart will be displayed for anthropometrics characteristics of the groups. The Shapiro wilk test will be used for assessing symmetrical data distribution. In case of symmetrical distribution following test will be used. The T independent test will be used to compare the mean of two groups. The paired T test will be used to compare the mean of pre and post treatment in both groups. In case of non symmetrical distribution the Wilcoxons test will be used to compare means of pre and post treatments in both groups, and Mann- whitney test will be used for comparing means of two different groups. The ANOVA with posthoc will be used to assess the changes in different measurement. The significance level for examining difference between two groups will be 0.05.

Sample template for the CONSORT diagram showing the flow of participants through each

stage of a randomized trial. The text boxes can be modified by clicking on them.

Assessed for eligibility

(n = …)

Excluded (n = …)

Not meeting inclusion criteria

(n = …)

Refused to participate

(n = …)

Other reasons (n = …)

Randomized (n = …)

Allocated to intervention

(n = …)

Received allocated

intervention (n = …)

Did not receive allocated

intervention (n = …)

(give reasons)

Allocation

EnrolCONSORT DIAGRAM

Assessed for eligibility

(n = …)

Excluded (n = …)

Not meeting inclusion criteria

(n) = …

Refused to participate

(n = …)

Other reasons (n = …)

# Enrollment

Randomized (n = 68 )

Allocation

Allocated to intervention

(n = 34)

Received allocated intervention (n = …)

Did not receive allocated intervention (n = …)

(give reasons)

Allocated to intervention

(n = 34)

Received allocated intervention (n = …)

Did not receive allocated intervention (n = …)

(give reasons)

**Follow up**

# Analysis

Lost to follow up

(n = …) (give reasons)

Discontinued intervention (n = …) (give reasons)

Lost to follow up

(n = …) (give reasons)

Discontinued intervention (n = …) (give reasons)

Analyzed (n = …)

Excluded from analysis

(n = …) (give reasons)

Analyzed (n = …)

Excluded from analysis

(n = …) (give reasons)

**GANTT CHART**

|  | **Months** | | | | | | | | | | | | | | | | | | | | | | | |
| --- | --- | --- | --- | --- | --- | --- | --- | --- | --- | --- | --- | --- | --- | --- | --- | --- | --- | --- | --- | --- | --- | --- | --- | --- |
| **Activity** | 1 | 2 | 3 | 4 | 5 | 6 | 7 | 8 | 9 | 10 | 11 | 12 | 13 | 14 | 15 | 16 | 17 | 18 | 19 | 20 | 21 | 22 | 23 | 24 |
| **Data collection** |  |  |  |  |  |  |  |  |  |  |  |  |  |  |  |  |  |  |  |  |  |  |  |  |
| **Data analysis and interpretation** |  |  |  |  |  |  |  |  |  |  |  |  |  |  |  |  |  |  |  |  |  |  |  |  |
| **submission** |  |  |  |  |  |  |  |  |  |  |  |  |  |  |  |  |  |  |  |  |  |  |  |  |

**CONSENT FORM IN ENGLISH**

**Description of the Research and Your Participation**

**You are invited to participate in a research study****. The purpose of this research is to evaluate the “Effects of breathing reeducation on cervical and pulmonary outcomes in chronic neck pain”.**

**Risks and Discomfort**

There are not known risks associated with this research.

**Potential Benefits**

Your participation will be beneficial in terms of treating chronic neck pain and its associated dysfunctions.

**Protection of Confidentiality**

We will do everything we can to protect your privacy. Your identity will not be revealed in any publication resulting from this study.

**Voluntary Participation**

Your participation in this research study is voluntary. You may choose not to participate and you may withdraw your consent to participate any time. You will not be penalized in any way should you decide not you participate or to withdraw from this study.

**CONSENT**

**I have read this consent form and have been given the opportunity to ask questions. I give my consent to participate in this study.**

Participant’s Signature __________________ Date: __________________

تحقیق میں شرکت کا دعوت نامہ **شمولیت کی دعوت دیتا /دیتی ہوں**

**عنوان: دایمی گردن درد کے مریضوں ميں کلينيکل نتا ءج پر سا نس لينے کے ا عادہ کے اثرات**

**نقصانات اور تکلیف: اس تحقیق** سے کسی قسم کے نقصان یا تکلیف کا اندیشہ نہیں ہے ۔

**ممکنہ فوائد:** آپکو ایک اہم تحقیق میں حصہ لینے کا موقعہ دیا جاۓ گا۔

**رازداری کا تحفظ:** ہم آپ کی معلومات کے تحفظ کے لیے وہ سب کچہ کریں گے جو ہم کر سکتے ہیں۔ تحقیق کے متعلق اکٹہی کیی گيی تمام معلومات کو انتہا ئی خفیہ رکھا جاے گا۔ ڈیٹا انٹری اور تجزیے کے دوران آپ کے متعلق وہ تمام معلومات جن سے آپ کی شناخت ہو سکتی ہو کو ختم کر دیا جاے گا۔ اس تحقیق کے نتیجے میں شائع ہونے والی کسی بھی اشاعت میں آپ کی شناخت کو ظاہر نہیں کیا جاے گا۔

**رضاکارانہ شمولیت:** اس تحقیقی مطالعہ میں آپ کی شرکت رضاکارانہ ہے۔ آپ کو شرکت نہ کرنے اور کسی بھی وقت پغیر وجہ بتانے اس تحقیق میں شمولیت کو چھوڑنے کا اختیار ہے۔ شرکت نہ کرنے یا اس میں شمولیت کو چھوڑنے کی صورت میں آپ کے خلاف کوئی کاروايی نہیں کی جاے گی

درجذیلمعلوماتتحقیقمیںشاملہونےوالوںکےلیےپڑھیںاورانکاجوابدیےگیےخانوںمیںدرجکریں

- میں نے معلوماتی شیٹ جو کہ تحقیق کی وضاحت کر رہی ہے کو سمجھ لیا ہےاورمجھے تحققیق کے سوالات کرنے کا موقع دیا گیا تھا۔
- میں سمجھ گیا/گيی ہوں کہ میری شرکت رضاکارانہ ہے اور یہ کہ میں کسی بھی وقت اپنا ارادہ بدل سکتا/سکتی ہوں اور تحقیق سے دستبردار ہو سکتا/سکتی
- میں سمجھ گیا/گیی ہوں کہ میرے جوابات خفیہ رکھے جاءیں کے۔ میں محقیقیين کو اس بات کی اجازت دیتا/دیتی ہوں کے وہ جوابات کو جانچ سکیں۔
- میں سممجھ گیا/گی ہوں کے معلومات میرے نام کے بجاے نمبر کی صورت میں محفوط کی جائيں گی۔ تا کہ میں نتائج کی اشاعت کے دوران کسی بھی طرح سے شناخت نہ کیا جا سکوں۔ میں اس بات سے رضامند ہوں کے جو معلومات مجھ سے لی جائہيں گی وہ تحقیق میں استعمال ہوں گی۔
- میں اوپر بتایی گی تحقیق میں شامل ہونے کے لیے رضامند ہوں اور محقیقین کو اپنا پتہ تبدیل ہونے کی صورت میں مطلع کروں گا/گی۔

رضا مندی:**ميں نے يہ اجازت نامہ پڑھا ہے اور مجھے سوال پوچھنے کا موقع ديا گيا ہے۔ ميں اس سٹڈی ميں شرکت کے راضی ہوں۔**

**شرکت کنندہ کا نام __________________ دستخط____________________ تاريخ ___________**

**اجازت لينے والے کا نام ________________ دستخط ____________________تاریخ ____________**

**اس اجازت نامہ کی ايک نقل آپکو دی جانی چاہے۔**

**
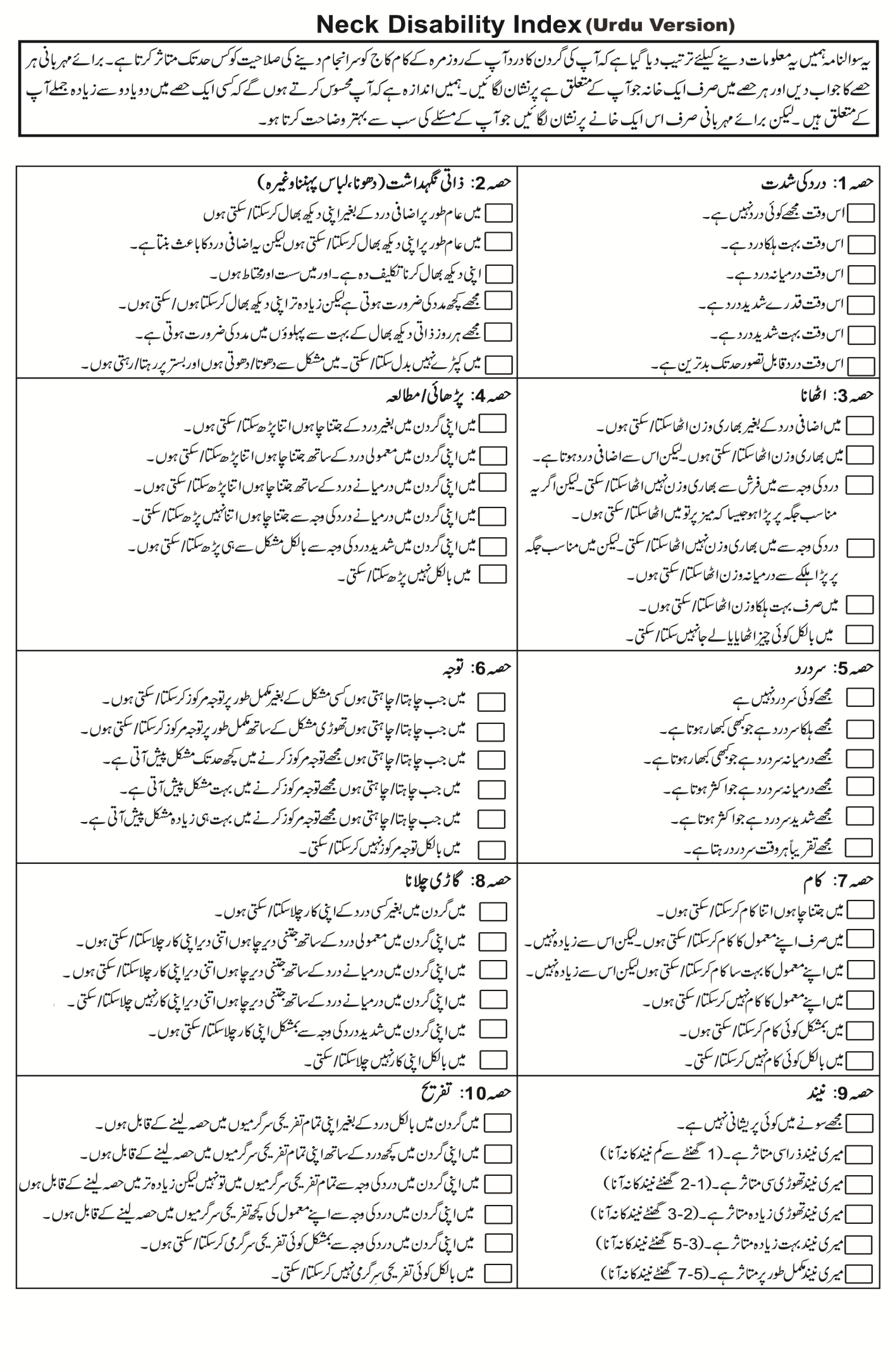
**

**REFRENCES**

1. Cohen SP, editor. Epidemiology, diagnosis, and treatment of neck pain. Mayo Clinic Proceedings; Elsevier;2015.

2. Hurwitz EL, Randhawa K, Yu H, Côté P, Haldeman S. The Global Spine Care Initiative: a summary of the global burden of low back and neck pain studies. European spine journal. 2018;27(6):796-801.

3. Nolet PS, Côté P, Kristman VL, Rezai M, Carroll LJ, Cassidy JD. Is neck pain associated with worse health-related quality of life 6 months later? A population-based cohort study. The Spine Journal. 2015;15(4):675-84.

4. Kim R, Wiest C, Clark K, Cook C, Horn M. Identifying risk factors for first-episode neck pain: A systematic review. Musculoskeletal Science and Practice. 2018;33:77-83.

5. Andersen LL, Hansen K, Mortensen OS, Zebis MK. Prevalence and anatomical location of muscle tenderness in adults with nonspecific neck/shoulder pain. BMC Musculoskeletal Disorders. 2011;12(1):169.

6. Jesus-Moraleida FRd, Pereira LSM, Vasconcelos CdM, Ferreira PH. Multidimensional features of pain in patients with chronic neck pain. Fisioterapia em Movimento. 2017;30(3):569-77.

7. Nicholas M, Vlaeyen JW, Rief W, Barke A, Aziz Q, Benoliel R, et al. The IASP classification of chronic pain for ICD-11: chronic primary pain. Pain. 2019;160(1):28-37.

8. Johnston V, Jull G, Darnell R, Jimmieson N, Souvlis T. Alterations in cervical muscle activity in functional and stressful tasks in female office workers with neck pain. European journal of applied physiology. 2008;103(3):253-64.

9. Kim JY, Kwag KI. Clinical effects of deep cervical flexor muscle activation in patients with chronic neck pain. Journal of physical therapy science. 2016;28(1):269-73.

10. Lascurain-Aguirrebeña I, Newham DJ, Galarraga-Gallastegui B, Critchley DJ. Differences in neck surface electromyography, kinematics and pain occurrence during physiological neck movements between neck pain and asymptomatic participants. A cross-sectional study. Clinical Biomechanics. 2018;57:1-9.

11. Jull GA, O'leary SP, Falla DL. Clinical assessment of the deep cervical flexor muscles: the craniocervical flexion test. Journal of manipulative and physiological therapeutics. 2008;31(7):525-33.

12. Dimitriadis Z, Kapreli E, Strimpakos N, Oldham J. Respiratory weakness in patients with chronic neck pain. Manual therapy. 2013;18(3):248-53.

13. de Zoete RM, Brown L, Oliveira K, Penglaze L, Rex R, Sawtell B, et al. The effectiveness of general physical exercise for individuals with chronic neck pain: a systematic review of randomised controlled trials. European Journal of Physiotherapy. 2019:1-7.

14. Koldaş Doğan Ş, Ay S, Evcik D. The effects of two different low level laser therapies in the treatment of patients with chronic low back pain: A double-blinded randomized clinical trial. Journal of back and musculoskeletal rehabilitation. 2017;30(2):235-40.

15. Blanpied PR, Gross AR, Elliott JM, Devaney LL, Clewley D, Walton DM, et al. Neck pain: revision 2017: clinical practice guidelines linked to the international classification of functioning, disability and health from the orthopaedic section of the American Physical Therapy Association. Journal of Orthopaedic & Sports Physical Therapy. 2017;47(7):A1-A83.

16. Beltran-Alacreu H, López-de-Uralde-Villanueva I, Fernández-Carnero J, La Touche R. Manual therapy, therapeutic patient education, and therapeutic exercise, an effective multimodal treatment of nonspecific chronic neck pain: a randomized controlled trial. American journal of physical medicine & rehabilitation. 2015;94(10S):887-97.

17. Radhakrishnan R, Senthil P, Rathnamala D, Gandhi PS. Effectiveness of global posture re-education on pain and improving quality of life in women with chronic neck pain. Int J Phys Educ Sports Health. 2015;1:7-9.

18. Kahlaee AH, Ghamkhar L, Arab AM. The association between neck pain and pulmonary function: a systematic review. American journal of physical medicine & rehabilitation. 2017;96(3):203-10.

19. Lustrin ES, Karakas SP, Ortiz AO, Cinnamon J, Castillo M, Vaheesan K, et al. Pediatric cervical spine: normal anatomy, variants, and trauma. Radiographics. 2003;23(3):539-60.

20. Falla D, Bilenkij G, Jull G. Patients with chronic neck pain demonstrate altered patterns of muscle activation during performance of a functional upper limb task. Spine. 2004;29(13):1436-40.

21. Kahlaee AH, Rezasoltani A, Ghamkhar L. Is the clinical cervical extensor endurance test capable of differentiating the local and global muscles? The Spine Journal. 2017;17(7):913-21.

22. Talia J. Appendix A: Muscles of respiration. Vocal Science for Elite Singers: a Tribute to the Unrelenting Pursuit of Excellence in the Art of Singing. 2017:263.

23. Woo S-D, Kim T-H, Lim J-Y. The effects of breathing with mainly inspiration or expiration on pulmonary function and chest expansion. Journal of physical therapy science. 2016;28(3):927-31.

24. Kapreli E, Vourazanis E, Strimpakos N. Neck pain causes respiratory dysfunction. Medical hypotheses. 2008;70(5):1009-13.

25. Tanigawa T, Nishimura N, Nakaoka H, Tsugitomi R, Okafuji K, Kitamura A, et al. D38 COPD: PHYSIOLOGIC EVALUATION: Clinical Risk Factors Of Decline In Forced Expiratory Volume In One Second Among Visitors With Obstructive Pulmonary Dysfunction Found In Medical Checkup. American Journal of Respiratory and Critical Care Medicine. 2016;193:1.

26. Kapreli E, Vourazanis E, Billis E, Oldham J, Strimpakos N. Respiratory dysfunction in chronic neck pain patients. A pilot study. Cephalalgia. 2009;29(7):701-10.

27. Dimitriadis Z, Kapreli E, Strimpakos N, Oldham J. Respiratory dysfunction in patients with chronic neck pain: What is the current evidence? Journal of bodywork and movement therapies. 2016;20(4):704-14.

28. Dimitriadis Z, Kapreli E, Strimpakos N, Oldham J. Hypocapnia in patients with chronic neck pain: association with pain, muscle function, and psychologic states. American journal of physical medicine & rehabilitation. 2013;92(9):746-54.

29. Dimitriadis Z, Kapreli E, Strimpakos N, Oldham J. Pulmonary function of patients with chronic neck pain: a spirometry study. Respiratory care. 2014;59(4):543-9.

30. Kim M-S, Cha Y-J, Choi J-D. Correlation between forward head posture, respiratory functions, and respiratory accessory muscles in young adults. Journal of back and musculoskeletal rehabilitation. 2017;30(4):711-5.

31. Zafar H, Albarrati A, Alghadir AH, Iqbal ZA. Effect of different head-neck postures on the respiratory function in healthy males. BioMed research international. 2018;2018.

32. Hyong I-H, Kim H-S, Lee S-Y. The effect of immediate pain and cervical ROM of cervical pain patients on stretching and manipulation. The Journal of Korean Physical Therapy. 2009;21.

33. Tozzi P, Bongiorno D, Vitturini C. Fascial release effects on patients with non-specific cervical or lumbar pain. Journal of bodywork and movement therapies. 2011;15(4):405-16.

34. Wirth B, Amstalden M, Perk M, Boutellier U, Humphreys B. Respiratory dysfunction in patients with chronic neck pain–Influence of thoracic spine and chest mobility. Manual therapy. 2014;19(5):440-4.

35. Jung J-h, Kim N-s. Relative activity of respiratory muscles during prescribed inspiratory muscle training in healthy people. Journal of physical therapy science. 2016;28(3):1046-9.

36. Kang J-i, Jeong D-K, Choi H. The effect of feedback respiratory exercise on muscle activity, craniovertebral angle, and neck disability index of the neck flexors of patients with forward head posture. Journal of physical therapy science. 2016;28(9):2477-81.

37. Wang H, Naghavi M, Allen C, Barber RM, Bhutta ZA, Carter A, et al. Global, regional, and national life expectancy, all-cause mortality, and cause-specific mortality for 249 causes of death, 1980–2015: a systematic analysis for the Global Burden of Disease Study 2015. The lancet. 2016;388(10053):1459-544.

38. Childs JD, Cleland JA, Elliott JM, Teyhen DS, Wainner RS, Whitman JM, et al. Neck pain: clinical practice guidelines linked to the International Classification of Functioning, Disability, and Health from the Orthopaedic Section of the American Physical Therapy Association. Journal of Orthopaedic & Sports Physical Therapy. 2008;38(9):A1-A34.

39. Smith BH, Fors EA, Korwisi B, Barke A, Cameron P, Colvin L, et al. The IASP classification of chronic pain for ICD-11: applicability in primary care. Pain. 2019;160(1):83-7.

40. de Koning CH, van den Heuvel SP, Staal JB, Smits-Engelsman BC, Hendriks EJ. Clinimetric evaluation of active range of motion measures in patients with non-specific neck pain: a systematic review. European spine journal. 2008;17(7):905-21.

41. En MCC, Clair DA, Edmondston SJ. Validity of the Neck Disability Index and Neck Pain and Disability Scale for measuring disability associated with chronic, non-traumatic neck pain. Manual therapy. 2009;14(4):433-8.

42. Cleland JA, Childs JD, Whitman JM. Psychometric properties of the Neck Disability Index and Numeric Pain Rating Scale in patients with mechanical neck pain. Archives of physical medicine and rehabilitation. 2008;89(1):69-74.

43. Farooq MN, Mohseni-Bandpei MA, Gilani SA, Hafeez A. Urdu version of the neck disability index: a reliability and validity study. BMC Musculoskeletal Disorders. 2017;18(1):149.

44. Juul T, Søgaard K, Roos EM, Davis AM. Development of a patient-reported outcome: the Neck OutcOme Score (NOOS)–Content and construct validity. Journal of rehabilitation medicine. 2015;47(9):844-53.

45. Debouche S, Pitance L, Robert A, Liistro G, Reychler G. Reliability and reproducibility of chest wall expansion measurement in young healthy adults. Journal of manipulative and physiological therapeutics. 2016;39(6):443-9.
